# A Mini-Review of the NADPH Oxidases in Vascular Dementia: Correlation with NOXs and Risk Factors for VaD

**DOI:** 10.3390/ijms18112500

**Published:** 2017-11-22

**Authors:** Dong-Hee Choi, Jongmin Lee

**Affiliations:** 1 Center for Neuroscience Research, Institute of Biomedical Science & Technology, Konkuk University, 120 Neungdong-ro, Gwangjin-gu, Seoul 143701, Korea; dchoi@kku.ac.kr; 2 Department of Medical Science, Konkuk University School of Medicine, 120 Neungdong-ro, Gwangjin-gu, Seoul 143701, Korea; 3 Department of Rehabilitation Medicine, Konkuk University School of Medicine, 120 Neungdong-ro, Gwangjin-gu, Seoul 143701, Korea

**Keywords:** oxidative stress (OS), vascular dementia (VaD), reactive oxygen species (ROS), NADPH oxidases (NOX), cognitive impairment, risk factors

## Abstract

Oxidative stress (OS) is one of the factors that cause dementia conditions such as Alzheimer’s disease and vascular dementia (VaD). In the pathogenesis of VaD, OS is associated with risk factors that include increased age, hypertension, and stroke. Nicotinamide adenine dinucleotide phosphate (NADPH) oxidases (NOXs) are a molecular source of reactive oxygen species (ROS). According to recent studies, inhibition of NOX activity can reduce cognitive impairment in animal models of VaD. In this article, we review the evidence linking cognitive impairment with NOX-dependent OS, including the vascular NOX and non-vascular NOX systems, in VaD.

## 1. Introduction

Vascular dementia (VaD) is the second most frequent type of dementia and accounts for 17% to 25% of all cases of dementia worldwide [1,2,3]. It is recognized by the common symptoms of cognitive function decline including reasoning, executive functions, memory, language, perception, and knowledge and cerebrovascular pathologies [4,5,6]. Although the exact etiopathogenesis of VaD remains unknown, the evidence reported to date seems to indicate that it has multiple causes including cerebrovascular disease and coexisting vascular risk factors such as aging, hypertension, atherosclerosis, and stroke [7,8,9,10]. Growing evidence indicates that atherosclerotic pathologies of the vasculature are a hallmark of the development of VaD [4], and that oxidative stress (OS) plays a causative role in atherosclerosis and other cardiovascular diseases [11]. Many of the risk factors of cerebrovascular disease and VaD also involve elevated OS [12]. 

**Figure 1:**
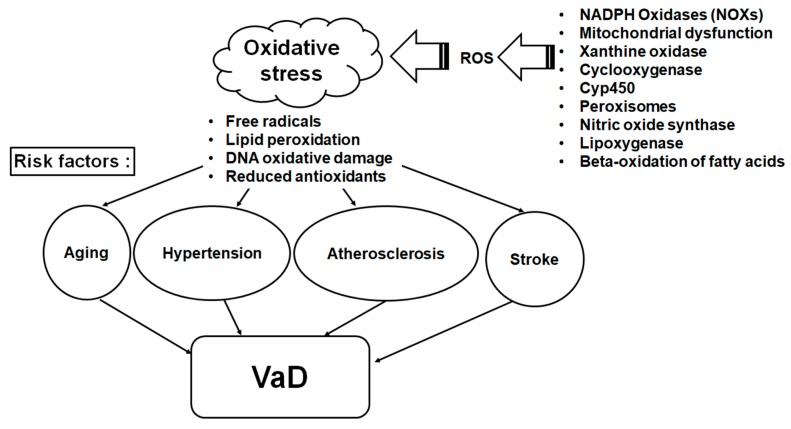
ROS produced by NOX in risk factors for vascular dementia. The open arrows indicate the induction of oxidative stress by ROS producing enzymes. The filled arrows indicate that the oxidative stress affects risk factors for VaD and then induces VaD. VaD: vascular dementia; ROS: reactive oxygen species; NOX: nicotinamide adenine dinucleotide phosphate (NADPH) oxidase.

OS imbalances the ratio of antioxidants and reactive oxygen species (ROS) resulting in damage to vessel endothelia, glial and neuronal cells, leading to neurovascular uncoupling and further cerebral blood flow reduction [6,13]. The overproduced ROS can destroy mitochondrial function and further induce hypoxia and OS [6,14]. The brain has multiple sources of ROS; however, it has been shown that membrane-bound nicotinamide adenine dinucleotide phosphate (NADPH) oxidase (NOX) enzymes are the primary sources of ROS during aging, hypoperfusion, stroke and hypertension [15]. ROS produced by NOX, which represent a major ROS-generation system, are often associated with the increased OS observed in the VaD and its risk factors [16,17]. Figure 1 shows the risk factors and NOXs of the ROS dependent mechanism for VaD.

Recent experimental studies have demonstrated that the inhibition of NOX activity attenuated chronic cerebral hypoperfusion-induced cognitive impairment in a rodent model [18,19]. In this review, we examined the current state of research regarding the roles of NOX in cognitive decline in VaD. In addition to NOX in VaD, we discussed the link between NOX and cognitive impairment in non-vascular cognitive disorders. To determine the association of NOX in cognitive impairment a keyword search on PubMed using the terms “cognitive impairment NADPH oxidase” yielded 76 citations, regardless of the year of publication. Search details of “cognitive impairment NADPH oxidase” was “cognitive dysfunction” or “cognitive” and “dysfunction” or “cognitive dysfunction” or “cognitive” and “impairment” or “cognitive impairment” and “NADPH oxidase” or “NADPH” and “oxidase” or “NADPH oxidase”. We excluded 16 review articles, two articles for cell studies, 11 articles included indirect correlation with NOX and cognitive function, and 12 articles with unmeasured cognitive function. We searched the reference lists of articles identified by this search strategy and selected those we judged relevant. Thirty-five publications were selected for this research. 

## 2. Association between Vascular Dementia (VaD) and Risk Factors and Oxidative Stress (OS)

In general, the risk factors for VaD are similar to those reported for cardiovascular disease and stroke. The risk factors for the development of dementia after stroke are multifactorial and contain age rising, vascular risk factors, stroke region, stroke incidence, global brain atrophy, and medical temporal lobe atrophy [20,21,22]. Comparable risk factors have been determined for VaD without stroke incidence, most notably increasing age and vascular risks, such as high cholesterol, hypertension, and atherosclerosis [20,23].

OS is caused by an environment where imbalance between the production of ROS and removal of ROS by antioxidant species is linked to the pathogenesis of dementia [24]. An enhanced level of ROS in the vasculature, suppressed nitric oxide bioactivities, and abnormal endothelial function resulting to vascular disease, are associated with VaD [24]. Although studies measuring markers of OS specifically in VaD are limited, there are data supporting a role for OS in this form of dementia, with most studies performed to date investigating markers of OS in the circulation [24]. Those studies reported a reduction in plasma antioxidant levels [25], elevation of plasma lipid peroxidation [26,27], and increased DNA oxidation in the cerebrospinal fluid [28] of patients with VaD. Regarding OS, it is linked to its risk factors as well as to VaD. Several reports have demonstrated that OS is involved in the pathogenesis of diabetes [29] and hypercholesterolemia [30]. Moreover, it has been reported that mitochondrial-induced OS is involved in hypertension-related vasculature damage [31], and that OS is responsible for the oxidation of low-density lipoproteins in atherosclerosis [32,33] and for the high levels of lipid hydroperoxides observed after ischemic stroke [16,34].

## 3. NADPH Oxidase in Cerebrovascular Disease 

### 3.1. NADPH Oxidase—An Overview

The family of NAPDH oxidases (NOXs) enzymes is a major source of ROS generation in various tissues [18]. These enzymes are capable of transporting electrons across the plasma membrane and producing superoxide [35]. The phagocytic NOX oxidase (gp91phox or NOX2) was the first identified in phagocytes [36] and is in charge of host defense via the release of considerable amounts of superoxide into phagosomes [18]. Seven NOX isoforms consisting of NOX1, NOX2, NOX3, NOX4, NOX5, dual oxidase (DUOX)1, and DUOX2, have been expressed in a variety of tissues, including brain [18,37]. 

The NOX and DUOX isoforms have similar binding sites for heme, flavin adenine dinucleotide, and NADPH [38,39] within each isoform and six conserved transmembrane α-helices [35]. Both DUOX 1 and DUOX2 contain an additional transmembrane α-helix, followed by a domain that is homologous to peroxidases [35]. The NOX and DUOX enzymes are differentially expressed and their regulation depends on the pathophysiological state of the body and brain [40].

NOX1 and NOX2 assemble with p22phox, and the full activation of p22phox with NOX1 and NOX2 need to bind with other regulatory subunits, p47phox (or its homolog NOX organizer 1, NOXO1), p67phox (or the homologous NOX activator 1, NOXA1), and Ras-related C3 botulinum toxin substrate (Rac) [41,42,43,44]. NOX4 has intrinsic activation and affinity with p22phox, but is not essential to other subunits for activation. NOX5 activation is regulated in a calcium–calmodulin-dependent manner, but not need any other subunit [41,43,44].

### 3.2. NOX-Dependent Generation of ROS Involves in Cerebrovascular Impairment

Recently, four isoforms (NOX1, 2, 4, and 5) of NADPH oxidases were discovered in vascular cells and have been shown to play a leading role in various physiological processes, including endothelial function, vascular tone, and vessel generation, as well as in vascular pathological conditions such as hypertension and stroke [45,46]. The activity of NOX in cerebral arteries is much higher than it is in the peripheral arteries [46]. Several studies have reported that NOX1, NOX2, and NOX4 manifested in cerebral arteries [47,48,49,50]. Kazama et al. reported that the NOX2 protein was expressed in endothelial and adventitial cells of cerebral resistance arteries [50]. Miller et al. found that the NOX4 protein was very highly (≥10-fold higher) expressed in rat basilar arteries when compared with the aorta, carotid, and mesenteric arteries [48]. Ago et al. [49] also showed that mRNA for NOX1, NOX2, and NOX4 were expressed in rat basilar endothelial and vascular smooth muscle (VSM) cells. The expression results, ranked in ascending order, are as follows: NOX2 < NOX1 < NOX4. They also demonstrated that the levels of mRNA or protein for NOX1 in endothelial cells were greater than in the VSM cells of basilar arteries [49]. The fine-tuned balance between the NOX-dependent generation of ROS and their detoxification by dismutase enzymes is a very delicate process [45]. The alteration of this balance in disease states result in either an over- or underproduction of ROS induced by vascular damage [45]. Several studies have highlighted the involvement of NOX isoforms in the pathophysiological process of vascular disease [51]. Increased expression of NOX subtypes is determined in various conditions including hypertension [47], insulin resistance [52], diabetes [53,54], and aging [55], ischemic stroke [56], and subarachnoid hemorrhage [57]. In particular, NOX1 and NOX2 have been shown to play deleterious roles [45], whereas NOX4 potentially plays a protective role in vascular diseases such as atherosclerosis [58]. Hence, increasing alteration of NOX expression arises in the status of pathophysiology. Subsequently enhancing of superoxide production would be expected through the increase of NOX activation in variety of disease [51].

## 4. NADPH Oxidase in Risk Factors for VaD

### 4.1. Increased NOX Expression is Associated with Increasing Age

Increased NOX activity is related to various cerebrovascular diseases and their risk factors. Cerebrovascular disease is thought to become more common with increasing age as older patients have an increased incidence of ischemic stroke and vascular cognitive impairment affects older persons [59,60]. Ali et al. have reported that the increased NOX-derived production of ROS induced learning and memory impairments observed in aged female rats [61]. Resting cerebral blood flow is reduced and cerebral circulation is dysregulated in older individuals [59,62]. These cerebrovascular effects are linked to the enhanced production of ROS in neuronal cells and cerebral vasculature [63]. Park et al. [64] reported that NOX-dependent ROS generated in neurons and blood vessels could involve the cerebrovascular disruption in older mice. Those ROS production and cerebrovascular dysfunction in the brain of 12-month-old mice were reversed by the NOX peptide inhibitor gp91ds-tat [64]. Moreover, the increase in ROS production was not determined in aged NOX2(-/-) mice [64]. Thus, NOX2(-/-) mice are reduced the oxidative stress and cerebrovascular damage induced by advancing age [64]. These findings suggested that NOX2 is a pivotal source of the neurovascular OS that induces the harmful cerebrovascular effects that are associated with increasing age [64]. 

### 4.2. NOX in Hypertension

The detailed role of vascular NOX oxidases as one of the first pathologies of hypertension has previously been elucidated [65]. Angiotensin II (Ang II) plays a central role via the renin–angiotensin system in the vascular transformation related to hypertension [46], and is an important inducer of increased NOX-dependent superoxide generation in the vascular smooth muscle cells [66] and throughout the cerebral vasculature [67,68,69,70]. Ang II induces vascular disease by various mechanisms that lead to ROS-mediated damage and endothelial dysfunction [71]. Ang II is functionally linked to NOX1, NOX2, and diverse to NOX4 in the vasculature [44]. The expression of the catalytic subunits of NOX1, NOX2, and NOX4 and of the p22phox cytosolic subunit are increased by Ang II stimulation [65,72,73]. Ang II-activated NOX1 and NOX2 appear to be important in vascular smooth muscle cells (VSMCs) from large and small arteries, respectively, in humans [44,74,75,76]. Nguyen Dinh Cat et al. demonstrated that NOX4 expression was higher in endothelial cells than in VSMCs, in basal conditions [44]. Activation of NOXs is mediated through the binding of Ang II to the Ang II-angiotensin receptor type 1 [77]. Ang II-mediated NOX activation sequentially produces intracellular H_2_O_2_, which induces vascular hypertrophy [78].

Recent studies have demonstrated that NOX activation participates in the pathogenesis of hypertension and associated vascular dysfunction [79]. NOX2-deficient(-/-) mice do not exhibit cerebrovascular OS and are spared from the alterations in cerebral blood vessels and functional hyperemia induced by Ang II [50,80,81]. Moreover, a peptide that inhibits the gathering of NOX oxidase or a pharmacological inhibitor of this enzyme suppresses the ROS generation and cerebrovascular dysfunction caused by Ang II [80,81,82]. The detrimental role of NOX1 and NOX2 in the vascular system has been verified in studies of gene-specific deletion or overexpression, whereas the functional importance of NOX4 remains controversial [79]. Schröder et al. have shown that endogenous NOX4 protected the vasculature during femoral artery ligated ischemic stress in animal models [79,83,84]. They found that NOX4-deficient animals had angiotensin II–induced hypertrophic and proinflammatory aortic response. Deletion of NOX4 increased inflammatory activation and aortic media hypertrophy in response to angiotensin II and reduced activation of endothelial nitric oxide synthase, nitric oxide production, and the heme oxygenase-1 (HO-1) system. In contrast, genetic deletion of NOX1 attenuated angiotensin II–induced vascular hypertrophy [79,83,84]. However, the protective role of NOX4 may differ from its role in the brain where NOX4 has been shown to contribute to OS and the neurodegeneration linked to stroke and other pathological conditions [79,85]. Therefore, further studies are required to determine the exact role of NOX4 in cerebrovascular diseases.

### 4.3. NOX in Stroke

There are three major types of stroke: ischemic stroke, subarachnoid hemorrhage (SAH), and intracerebral hemorrhage (ICH) (Table 1) [86]. Both the depletion of oxygen during ischemia and the replenishment of oxygen during reperfusion result in the tissue damage observed in stroke [40]. Ischemia/reperfusion injury is related to increased levels of ROS. OS can arise from increased generation of ROS in the ischemia/reperfusion injury [87,88]. There is increasing evidence that NOX-medicated ROS contribute to the pathology that follows cerebral ischemia/reperfusion [35,89,90,91]. Increased activation of the NOX1 [92], NOX2 [92,93,94,95], and NOX4 [85,96] isoforms is involved in ROS production and the development of the pathological condition that follows cerebral ischemia [40]. The expression of NOX1, NOX2, and NOX4 after focal cerebral ischemia was assessed in various cell types, including neurons, astrocytes, and microglia [40]. Remarkably, superoxide production and NOX2 expression have also been shown to be increased in mouse cerebral arteries after focal cerebral ischemia [95]. NOX-oxidase-mediated ROS and Rac1 activation in the hippocampus contributes to cognitive impairment after cerebral ischemia [97,98]. Recent studies have suggested that NOX activation is associated with hippocampal cell death and cognitive impairment in rats with cerebral hypoperfusion [18,19,99]. A recent study reported that the upregulation of p47phox oxidase induces spatial memory deficits and hippocampal oxidative DNA damage in chronic intermittent hypoxia conditions [100]. This finding suggests that these factors contribute considerably to cerebral ischemia-induced vascular dysfunction and are risk factors for VaD [40,95]. 

There are two types of hemorrhagic stroke: SAH and ICH. SAH is one of the most destructive cerebrovascular diseases [86]. Increased NOX activation has also been associated with the pathology of SAH [40]. Animal studies have reported that elevation of superoxide occurred as early as 12 h after SAH [57] followed by the enhancement of the expression and activation of NOX2 (gp91phox) [101,102,103], p47phox [104], and Rac1 [57] in the rat cortex or striatum at 12–48 h after SAH. The most recent study in this area demonstrated that the levels of the NOX2 and NOX4 proteins were increased in the perilesional neurons and astrocytes in brain tissues from patients with SAH. In an SAH rat model, the NOX2 inhibitor gp91ds-tat and the NOX4 inhibitor GKT137831 decreased SAH-induced neuronal death and degeneration [105]. 

In a rat model of the other type of hemorrhagic stroke, ICH, the activity of NOX2 increased significantly in the striatum after ICH [103]. Increased expression of gp91phox (NOX2) was mainly observed in activated microglia in ICH-injured brains [106]. 

These findings indicated that NOX activation has influences the early stage of hemorrhagic stroke and the relatively prolonged stage of ischemic stroke [40].

## 5. Association of NADPH Oxidase in Cognitive Impairment

We focused on the involvement of the NOXs in the cognitive deficits of vascular and non-vascular diseases in this section. Emerging evidence demonstrates that NOX oxidases are involved in the cognitive dysfunction observed in various diseases and in vivo experiments (Table 2).

In cerebrovascular damaged models such as chronic hypoperfusion [18,97,98,99], cerebral ischemic reperfusion [107], and intermittent hypoxia [108,109,110,111] NOX activity and expression of NOX isoforms (NOX1, NOX2, p67phox, and p47phox) increased and cognitive functions were impaired. However, treatment of NOX inhibitor apocynin, ROS scavenger tempol, Rac GTPase inhibitor, downregulation of NOX1 mRNA, and gp91phox knockout reversed those impaired cognitive behaviors. Moreover, aged AD-related transgenic mice have increased NOX activities and expressions of NOX2 and NOX4 correlated with cognitive decline. Their mechanisms may involve oxidative stress and deposition of amyloid beta [112,113,114,115]. 

Furthermore, studies on cognitive deficits in non-vascular disease demonstrated that increased NOX2 and p47phox expression and activation was involved in cognitive impairment in traumatic brain injury (TBI) [116,117,118,119], various encephalopathies [120,121,122,123], and metabolic diseases [13,124,125]. Chandran at al. [116] reported that NOX2 increased and transcription factor Nrf2 that decreased ROS were induced after TBI. The combination therapy (NOX inhibitor apocynin + Nrf2 activator TBHQ) improved cognitive dysfunction in TBI [116]. Di Filippo et al., found that multiple-sclerosis-associated cognitive dysfunction was caused by synaptic dysfunction via a long-term potentiation (LTP) blockade mediated by NOX2 [120]. Ji et al. and Hernandes et al. showed that selective phenotype loss of interneurons mediated by NOX2 activation lead to cognitive impairments in a mouse model of sepsis-associated encephalopathy (SAE) [122,123]. Won et al. [124] confirmed the relationship between cognitive deficits and recurrent hypoglycemia in diabetic rats. They found that NOX-activity dependent oxidative damage and microglial activation increased in the dendritic region of hippocampal CA1 by hypoglycemia in diabetic rat. This study suggests that oxidative injury by hypoglycemia is linked to long term cognitive dysfunction in diabetic patients [124]. 

More interestingly, Ansari and Scheff showed that the overall NOX enzyme activity level and protein levels of p67phox, p47phox, and p40phox isoforms were elevated in the human postmortem brains of Alzheimer’s disease (AD) [126]. Another human study by Bruce-Keller at al. found significant elevations in NOX activity and gp91phox and p47phox expression in the temporal gyri of mild cognitive impairment (MCI) patients when compared with the controls [127]. The increased gp91phox and p47phox expression were shown in the microglia and neurons [127]. Human studies have suggested that an elevated NOX-associated redox pathway might contribute to AD progression [126,127].

## 6. Conclusions

VaD can result from a loss of brain cells induced by hypoperfusion via damage to the vascular system. The factors that can cause vascular damage in VaD include aging, hypertension, and stroke. NOX oxidases play a role as a major ROS generator in cerebrovascular and neurodegenerative diseases. NOX-mediated ROS induce neuroinflammation and OS in blood vessels in conditions of aging, hypertension, and stroke. Moreover, NOX activation has also been linked to cognitive impairment in various neurological disorders. In this review, we summarized the current research regarding the association between risk factors for VaD and NOX: (1) Increased NOX expression was associated with the aging risk factor; (2) NOX activation produced intracellular H_2_O_2_, which induced vascular hypertrophy as a hypertension risk factor; (3) Increased activation of the NOX1, NOX2, and NOX4 isoforms was involved in ROS production and the development of the pathological condition in stroke risk factor; and (4) NOXs associated cognitive impairment was involved not only in vascular dementia but also in non-vascular diseases. 

Considering the relationship between NOX and the risk factors for VaD (including aging, hypertension, and stroke), we suggest that further studies are necessary to examine the different roles of the vascular NOX isoforms and neuronal NOX isoforms in VaD. Moreover, we emphasize the contention that the regulation of NOX levels in cerebral vessels and the modulation of NOX activation in neurons and glial cells are important for the prevention and treatment of VaD. 

Therefore, regulation of NOX may serve as a potential therapeutic target for VaD. Furthermore, emerging translational studies for controlling the risk factors through NOX regulation in VaD may have serious impacts on the significance of clinical application.

## Figures and Tables

**Table 1:** Nicotinamide adenine dinucleotide phosphate (NADPH) oxidases (NOX) expressions in three major types of stroke.

Stroke Types	Ischemic Stroke	Subarachnoid Hemorrhage	Intracerebral Hemorrhage
NOX isoforms	NOX1, NOX2, NOX4	NOX2, NOX4	NOX2
Region Cell types	Cortex, hippocampus, cerebral arteries Neurons, astrocytes, microglia	Cortex, striatum neurons, astrocytes	Striatum Microglia

**Table 2:** Experiments of NOX expressions associated cognitive function.

Disease model	Increase NOX isoforms	Control of NOX	Mechanism of action	Reference
Two-vessel occlusion rat	gp91phox, p47phox, or p67phox	Apocynin ^1^	Oxidative stress	[98]
	NOXs activity, NOX1	Apocynin, shRNA NOX1 AAV	Oxidative stress	[18]
bilateral common carotid artery stenosis mice	NOXs activity	Apocynin	Oxidative stress	[107]
	p67phox	Tempol ^2^	Oxidative stress	[99]
cerebral ischemic reperfusion rat	NOXs activity	Rac GTPase inhibitor (NSC23766)	Oxidative stress	[97]
Obstructive sleep apnea, Long-term exposure to intermittent hypoxia (LTIH) mice	NOX2 (gp91phox), p47phox, p22phox	gp91phox(-/-), erythropoietin, Apocynin	Lipid peroxidation and oxidative DNA damage	[108,109,110,111]
Aged Tg2576 Mice	NOXs activity	Apocynin	Oxidative stress and cerebrovascular dysfunction	[112]
Aged APP and PS1 knock-in mice	NOXs activity NOX4	Young age	Deposition of Aβ1-42	[113]
Tg2576 and NOX2(-/-) mice	NOX2	NOX peptide Inhibitor gp91ds-tat NOX2(-/-)	ROS generation	[114]
gp91phox(-/-) and IL-6(-/-) aged mice	NOXs activity, NOX2	Apocynin, gp91phox(-/-), IL-6(-/-)	Oxidative stress	[115]
ACE2KO mice	p22phox, p40phox, p67phox, and gp91phox	angiotensin (Ang)-converting enzyme (ACE), Tempol	Oxidative stress	[128]
Renin/angiotensinogen transgenic mice	p47phox and Nox4	Tempol	Oxidative stress	[129]
TBI	NOX2	Apocynin, NOX2(-/-)	ROS formation	[116]
Moderate lateral fluid percussion injury mice	NOXs activity	bradykinin receptors B_2_ antagonist (HOE-140), Apocynin	ROS formation	[117,118]
Post-traumatic stress disorder	NOX2	Environmental enrichment	Oxidative stress	[119]
Encephalomyelitis model of multiple sclerosis	NOX2	Minocycline ^3^, apocynin	hippocampal synaptic plasticity deficit	[120]
Sepsis-associated encephalopathy	NOX2	Apocynin, gp91phox(-/-)	Inflammation and oxidative stress	[122,123]
Minimal hepatic encephalopathy rats	p47phox	Apocynin	ROS formation	[121]
Recurrent/moderate hypoglycemia rat	NOXs activity	Apocynin	Oxidative damage	[124]
Streptozotocin diabetes induced vascular dementia in rats	NOXs activity	NOX inhibitor, 4′-hydroxy-3′-methoxyacetophenone	Oxidative stress	[130]
Endoplasmic reticulum stress, domoic acid-treated mice	p47phox and gp91phox	estrogen receptor-α	ROS formation, ER stress	[131]
Sleep fragmentation in mice	gp91phox	gp91phox(-/-) mice	Lipid peroxidation and oxidative DNA damage	[132]
Postoperative aging mice	NOX2	Apocynin,	Oxidative stress	[133,134]
Ketamine treated rats	NOX2	Apocynin	Oxidative damage	[135]
Sevoflurane exposure mice	p22 phox	Apocynin	Increasing superoxide concentrations	[136]
Chronic granulomatous disease mice	gp91phox and p47phox	diphenylene iodonium or apocynin gp91phox and p47phox KO	LTP Blocking	[137]
p47phox and nNOS double KO mice	p47phox	p47phox(-/-)and nNOS(-/-)	ROS and NO formation	[138]
AD postmortem brains	p67phox, p47phox, and p40phox		Increasing of redox pathways	[126]
MCI postmortem brains	NOXs activity, gp91phox, p47phox		Microglia activation	[127]

^1^ NOX inhibitor, Apocynin; ^2^ ROS scavengers, Tempol; ^3^ Minocycline, antibiotic. MCI: mild cognitive impairment; KO: knockout; ACE: angiotensin (Ang)-converting enzyme.

## Abbreviations

NADPH	Nicotinamide adenine dinucleotide phosphate
OS	Oxidative stress
VaD	Vascular dementia
ROS	Reactive oxygen species
NOX	NADPH oxidase
phox	Phagocytic oxidase
DUOX	Dual oxidase
NOXO1	NOX organizer 1
NOXA1	NOX activator 1
Rac	Ras-related C3 botulinum toxin substrate
Ang II	Angiotensin II
SAH	Subarachnoid hemorrhage
ICH	Intracerebral hemorrhage
CA1	Cornu Ammonis 1
ACE	Angiotensin (Ang)-converting enzyme
IL-6	Interleukin-6
nNOS	Neuronal nitric oxide synthase
TBI	Traumatic brain injury
TBHQ	Tertiary Butylhydroquinone
LTP	Long-term potentiation
SAE	Sepsis-associated encephalopathy
AD	Alzheimer’s disease
KO	Knockout
Nrf2	NF-E2-related factor-2
